# Exploiting parameter space in MOFs: a 20-fold enhancement of phosphate-ester hydrolysis with UiO-66-NH_2_ †
†Electronic supplementary information (ESI) available. See DOI: 10.1039/c4sc03613a



**DOI:** 10.1039/c4sc03613a

**Published:** 2015-02-24

**Authors:** Michael J. Katz, Su-Young Moon, Joseph E. Mondloch, M. Hassan Beyzavi, Casey J. Stephenson, Joseph T. Hupp, Omar K. Farha

**Affiliations:** a Department of Chemistry , Northwestern University , 2145 Sheridan Road , Evanston , Illinois 60208 , USA . Email: j-hupp@northwestern.edu ; Email: o-farha@northwestern.edu; b Chemical Science and Engineering Division , Argonne National Laboratory , 9700 S. Cass Avenue , Argonne , Illinois 60439 , USA; c Department of Chemistry , Faculty of Science , King Abdulaziz University , Jeddah , Saudi Arabia

## Abstract

The hydrolysis of nerve agents is of primary concern due to the severe toxicity of these agents. Using a MOF-based catalyst (UiO-66), we have previously demonstrated that the hydrolysis can occur with relatively fast half-lives of 50 minutes. However, these rates are still prohibitively slow to be efficiently utilized for some practical applications (*e.g.*, decontamination wipes used to clean exposed clothing/skin/vehicles). We thus turned our attention to derivatives of UiO-66 in order to probe the importance of functional groups on the hydrolysis rate. Three UiO-66 derivatives were explored; UiO-66-NO_2_ and UiO-66-(OH)_2_ showed little to no change in hydrolysis rate. However, UiO-66-NH_2_ showed a 20 fold increase in hydrolysis rate over the parent UiO-66 MOF. Half-lives of 1 minute were observed with this MOF. In order to probe the role of the amino moiety, we turned our attention to UiO-67, UiO-67-NMe_2_ and UiO-67-NH_2_. In these MOFs, the amino moiety is in close proximity to the zirconium node. We observed that UiO-67-NH_2_ is a faster catalyst than UiO-67 and UiO-67-NMe_2_. We conclude that the role of the amino moiety is to act as a proton-transfer agent during the catalytic cycle and not to hydrogen bond or to form a phosphorane intermediate.

## Abstract 2

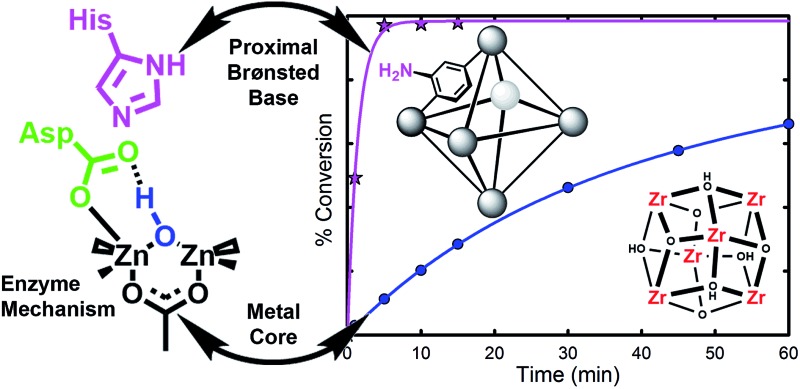
Using the enzymatic mechanism of phosphoesterase as a template, we were able to modify a metal–organic framework such that the hydrolysis rates were 50 times faster than previously demonstrated with UiO-66.

## Introduction

**Fig. 1:**
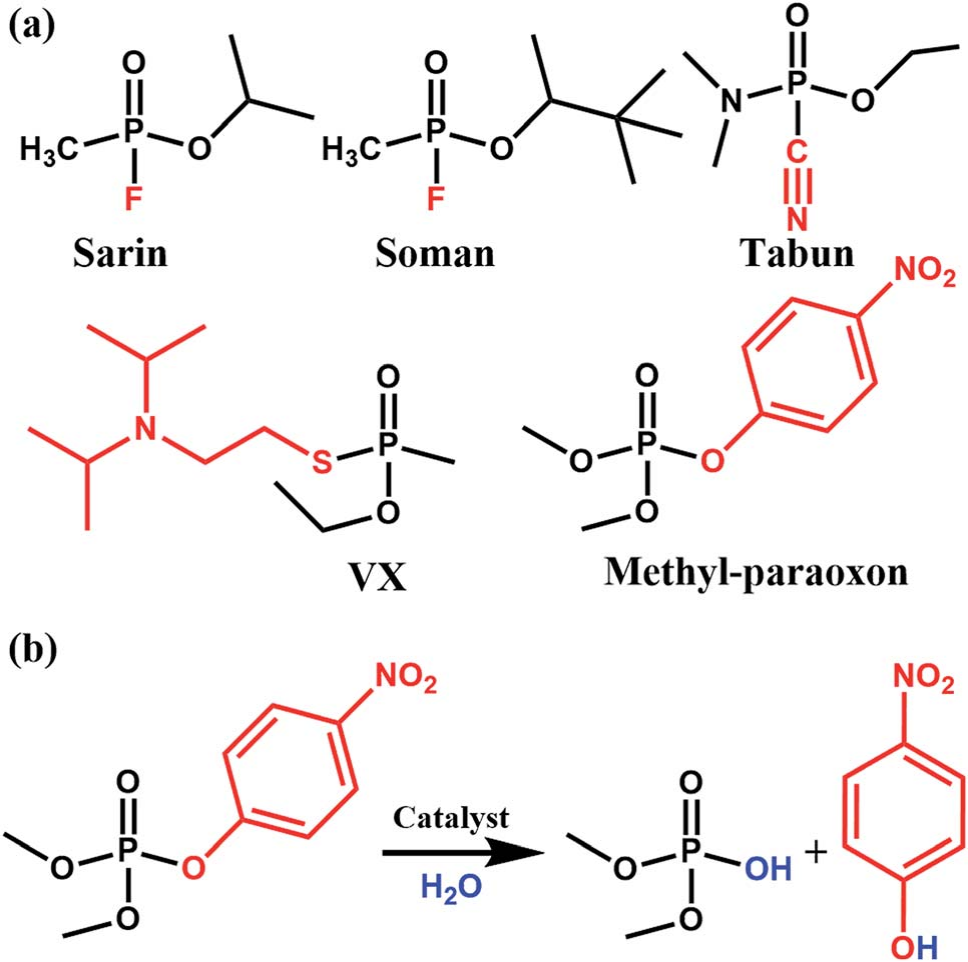
(a) Molecular structure of various nerve agents as well as the pesticide methyl-paraoxon. The fragment shown in red is the desired leaving group during hydrolysis. (b) Catalytic hydrolysis reaction for the hydrolysis of methyl-paraoxon.

Phosphate-based compounds are key ingredients in biological systems. The cleavage and formation of P–O bonds are responsible for converting ATP to ADP and *vice versa*.^1,2^ It is thus not surprising that organophosphorous compounds such as the ones shown in Fig. 1 are capable of disrupting biological functions.^2–5^ Sarin, for example, is a phosphate-based chemical warfare agent that has unfortunately shown resurgence in recent times.^4,6,7^ The P–F bond is easily cleaved followed by the formation of a strong acetylcholinesterase inhibitor due to the favorable P–O bond formed; this often leads to asphyxiation.^6,8^

Other organophosphorous containing compounds, such as methyl-paraoxon, are phosphate-based pesticides. Methyl paraoxon's toxicity is due to the ease in which the P–O group of the nitrophenol can undergo transesterification under physiological conditions. Interestingly, due to the high-use of these pesticides, enzymes have evolved as a response to these pesticides. Ultimately, this has led to enzymes, both natural and synthetic, which have a high specificity^9^ and efficacy towards this family of nerve agents. Thus, given the importance of phosphate esters in biological systems, it is not surprising that there has been much work done in studying enzymes, proteins, and small molecules which are capable of performing phosphate-ester hydrolysis on nitrophenol-containing pesticides/nerve-agent simulants.

Transition-metal, or rather Lewis-acid, hydrolysis is ubiquitous in the field of phosphate-hydrolysis.^10–16^ Taking a lesson from nature, many transition-metal complexes employ hydroxide bridged dimers as catalysts.^10,11,17–20^ For example, the family of phosphoesterase enzymes utilize a Zn^II^–OH–Zn^II^ active site which is often further supported by a bridging carboxylate.^21^ Given that early row transition metals are often found in biological materials due to bioavailability, researchers such as Brown have utilized coordination complexes with more Lewis-acidic centres such as La^III^ and Zr^IV^ as catalysts for phosphate-ester hydrolysis.^11,14,22^

Our group has also been investigating the role of Lewis-acidic centres such Al^III^ and Zn^II^ as catalysts for the degradation of phosphates such as methyl-paraoxon.^23–26^ We observed that Al^III^–porphyrin dimers^24^ and tetramers^26^ significantly outperformed their Zn^II^ counterparts; half-lives of 10 hours were observed for the methanolysis of methyl- and phenyl-paraoxon by these Al^III^-complexes. In-line with these materials, we began looking at porous-organic polymers (POPs) as potential catalysts.^23,25^ These POPs offered the combined advantage of homogeneous catalysis (*e.g.*, well defined catalysts such as Al^III^ porphyrins^23^ and La^II^ catecholates^25^) and heterogeneous catalysis (*e.g.*, insoluble catalyst that can be easily removed).

We observed two key design features in the aforementioned systems: (i) strong Lewis-acidic metals were key for fast turn over, and (ii) the use of simple synthesis, such as those used in the formation of POPs, are attractive heterogeneous catalysts. Thus, we turned our attention to another class of porous materials, namely, metal–organic frameworks (MOFs).

**Fig. 2:**
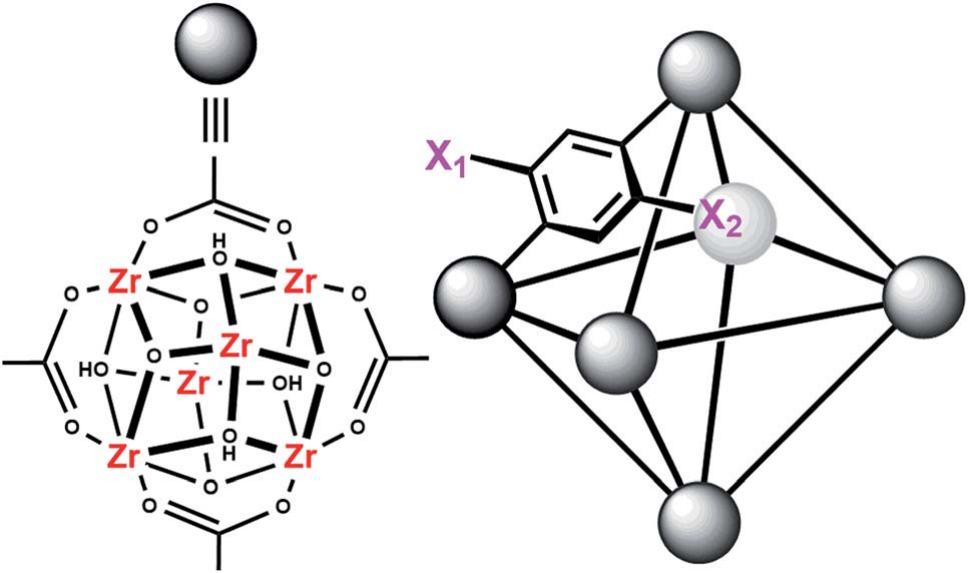
Molecular structure of the node of UiO-66 showing four of the twelve bound carboxylates (left) and the connectivity of the octahedral pore in UiO-66 (right). X_1_ = X_2_ = H for UiO-66, X_1_ = NO_2_ and X_2_ = H for UiO-66-NO_2_, X_1_ = X_2_ = OH for UiO-66-(OH)_2_, and X_1_ = NH_2_ and X_2_ = H for UiO-66-NH_2_.

MOFs are porous materials formed from inorganic cationic nodes (*e.g.*, Zr_6_O_4_(OH)_4_^12+^) and anionic bridging ligands (*e.g.*, 1,4-benzenedicarboxylate).^27^ In addition to our two design principles above, MOFs are ordered/crystalline materials which are needed for rapid screening and hypothesis driven research. Due to their high porosity^28^ as well as thermal and chemical stability,^29–32^ MOFs are promising materials in applications such as gas-storage,^33–35^ chemical separations,^36–41^ sensing,^42,43^ catalysis,^44–47^ and light harvesting.^48–51^ In keeping with our aforementioned design principle, we have shown that UiO-66 (Fig. 2), a MOF containing highly Lewis-acidic Zr^IV^ ions, in the form of Zr^IV^_6_O_4_(OH)_4_^12+^ clusters, which can be easily formed in high yield using a variety of different synthetic procedures,^52^ had a half-life of 50 minutes for the hydrolysis of methyl-paraoxon at room temperature.^53^ This half-life is among the fastest, non-enzymatic, half-lives reported to date. Hatton *et al.* ^54^ have also demonstrated that the Lewis-acidic Cr^III^ centre in Cr-MIL-101 is also capable of hydrolysing phosphate esters with a half-life of nearly 3 hours.

**Fig. 3:**
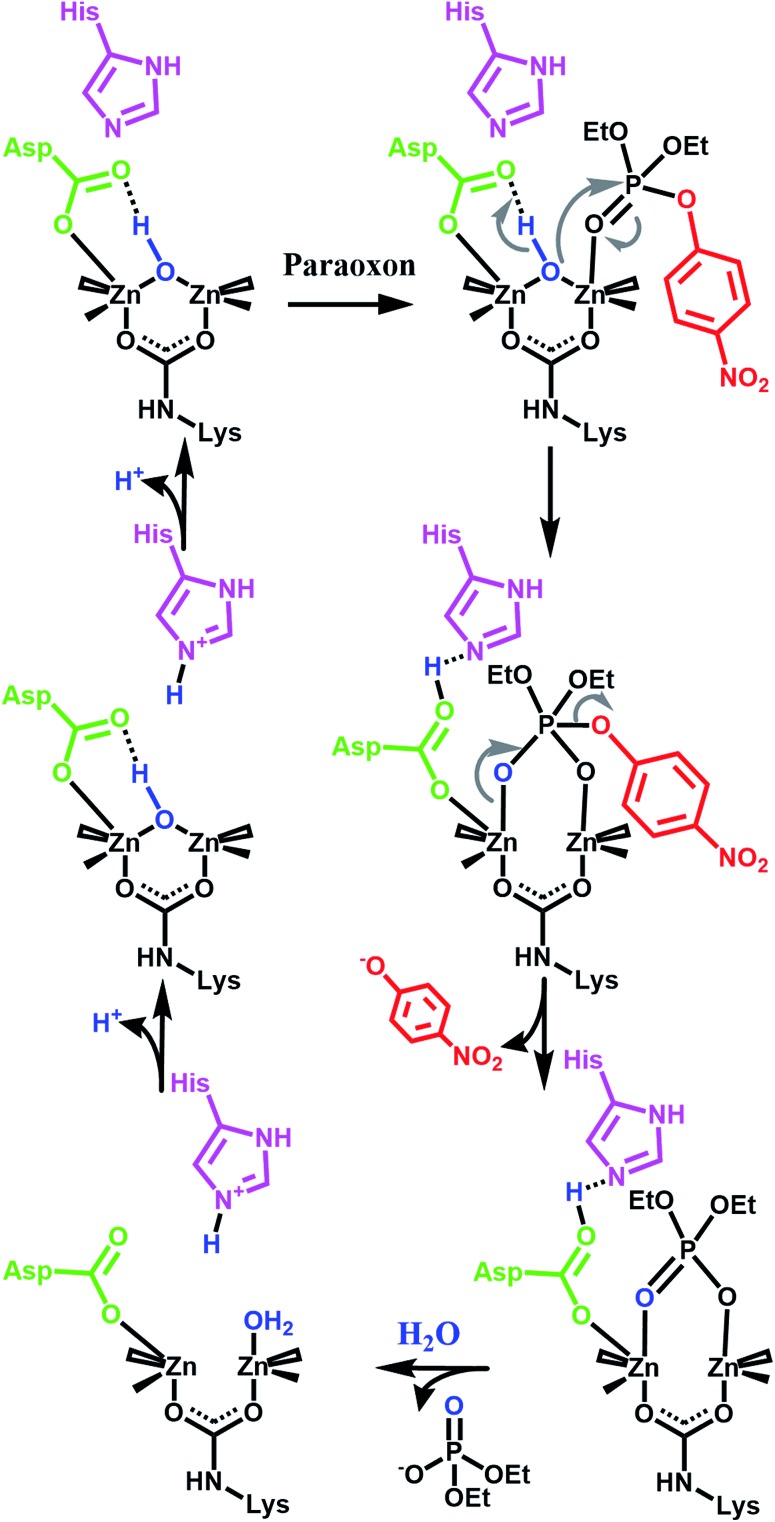
One proposed enzymatic mechanism by Raushel for the hydrolysis of paraoxon.^21^ Note the presence of proximal bases such as aspartate carboxylate and a histidine imidazole.

Our initial success with UiO-66 was based on the observation that enzymatic systems use bimetallic Lewis-acidic metal centres bridge by a hydroxide. Along the same line, we continue with the biomimetic approach by looking at the necessary components of the mechanism.^16,21,55–59^ Raushel, for example, has used a combination of experimental and computational chemistry to probe the mechanism (Fig. 3). They, and others, have shown the importance of a proximal aspartate and histidine.^60^ Both moieties act as a proximal base, transferring protons to and from these moieties at key portions of the catalytic cycle. In the absence of these moieties, the hydrolysis rates were diminished.

With this mechanistic insights in-mind, we hypothesized that UiO-66-NH_2_ would be a faster catalyst than UiO-66. To that end, we investigate the role of UiO-66-NH_2_ for its ability to enhance the reaction rate of our catalysis. As shown below, UiO-66-NH_2_ is capable of decreasing the half-life by roughly 20 fold.

## Experimental

All reagents were purchased from commercial sources and used without further purification. UiO-66, UiO-66-NH_2_, UiO-66-(OH)_2_, UiO-66-NO_2_, UiO-67, UiO-67-NMe_2_, UiO-67-NH_2_, and methyl-paraoxon were synthesized according to literature procedures (see the ESI† for powder X-ray diffractograms and N_2_ isotherms).^52,61^ UiO-67, UiO-67-NMe_2_, and UiO-67-NH_2_ were de-solvated by supercritical CO_2_.^26,52^

**Fig. 4:**
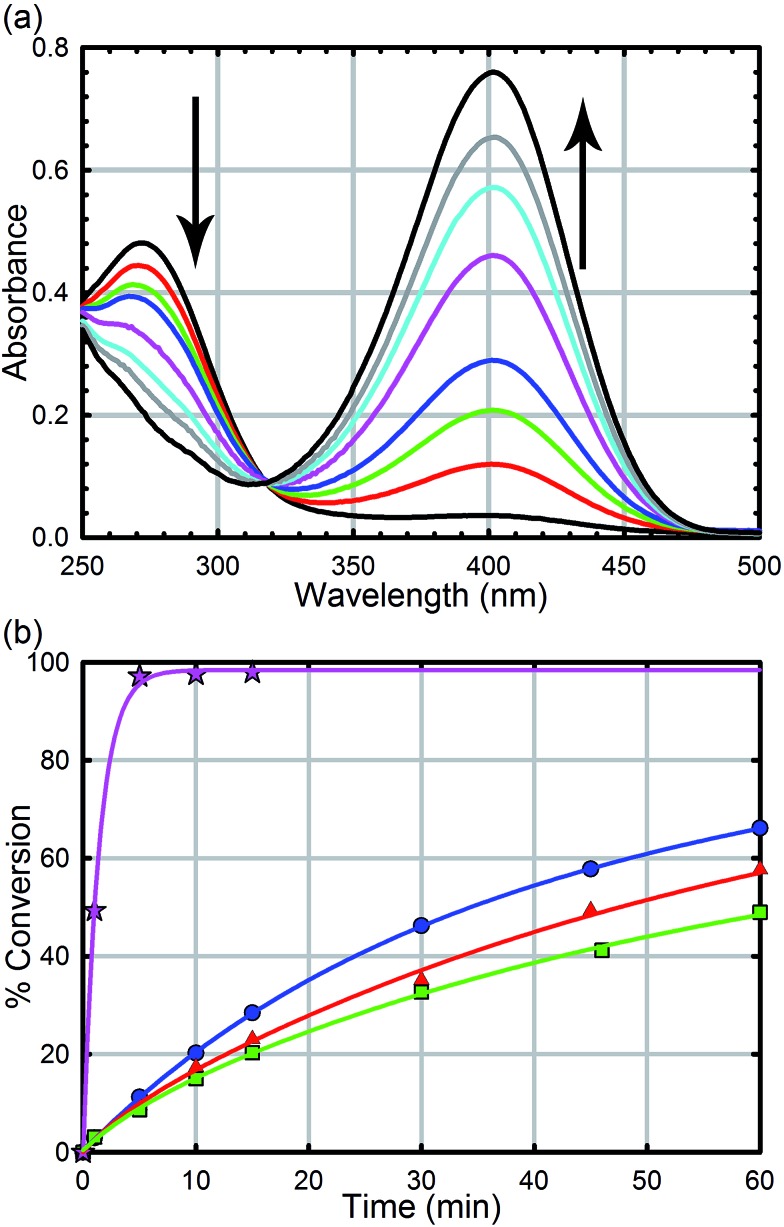
(a) UV-Vis trace of the hydrolysis of methyl-paraoxon as a function of time using UiO-66. The arrows denote the disappearance of the starting material at 275 nm and the appearance of the product at 407 nm. (b) Hydrolysis rate of UiO-66 (blue circles), UiO-66-NO_2_ (red triangles), UiO-66-(OH)_2_ (green squares), and UiO-66-NH_2_ (pink stars).

Hydrolysis experiments were carried out at room temperature as described previously. Briefly, to a solid sample of UiO-66 (2.5 mg, 6 mol%, 0.0015 mmol; 0.045 mol% of active surface sites) in an Eppendorf tube was added an aqueous solution of *N*-ethyl-morpholine (0.45 M); the *N*-ethyl-morpholine is used as a buffer to limit pH changes. The resulting mixture was stirred for 30 minutes to finely disperse the MOF particles. To the suspension was then added methyl-paraoxon (6.2 mg, 0.025 mmol). Periodic monitoring was carried out by removing a 20 μL aliquot from the reaction mixture and diluting with an aqueous solution of *N*-ethyl-morpholine (10 mL, 0.45 M) prior to UV-Vis measurements (Varian Cari 5000) (Fig. 4a). Progress of the reaction was monitored by following the *p*-nitrophenoxide absorbance at 407 nm to avoid overlapping absorptions with other species. No spectral evidence for the *p*-nitrophenol was observed at this pH (10.2). All background reactions were carried out under identical reaction conditions without the MOF catalyst.

Initial rates were determined using the method of initial rates.^62^ Polynomial fits of order 3–5 were used with the lowest observed correlation coefficient of 0.98.

## Results and discussion

As we have previously demonstrated, the hydrolysis of methyl-paraoxon with UiO-66 is the fastest MOF-based catalysts reported to date.^53^ The ease in which alternative linkers can be incorporated into the framework^29,31,52,63–72^ makes UiO-66 an attractive platform for generating a large parameter space which can be used to easily probe a mechanism. As illustrated in Fig. 4b and Table 1, just as the enzymatic hydrolysis is enhanced by the presence of a proximal anchored base, the hydrolysis rate of UiO-66-NH_2_ is 20 times greater than that of UiO-66.^73^ In order to compare the amino moiety in our system with the aspartate and histidine moieties in the enzymatic system, we also synthesized UiO-66-NO_2_ and UiO-66-(OH)_2_. These two MOFs are capable of hydrogen bond donating and receiving, similar to UiO-66-NH_2_, but they cannot act as a Brønsted base like the amino moiety. As illustrated in in Fig. 4 and Table 1, there is no significant difference between the initial rates of UiO-66-(OH)_2_ and UiO-66-NO_2_; the initial rates for the catalytic hydrolysis by UiO-66-(OH)_2_ and UiO-66-NO_2_ (0.0063–0.0070 mmol s^–1^ respectively) are slightly slower than that of UiO-66 (0.010 mmol s^–1^). Based on these observations, we concluded that the amino moiety acts as a base and not as a hydrogen-bonding moiety. Furthermore, considering there is no difference between UiO-66, UiO-66-NO_2_ (an electron withdrawing group) and UiO-66-(OH)_2_ (a steric electron donating group), there is little to no electronic effect and only a slight steric effect from the linker on the rate limiting step of the hydrolysis reaction (Table 1).

**Table 1:** Initial rates and turn-over frequencies (TOFs) for UiO-66, UiO-66-(OH)_2_, UiO-66-NO_2_, UiO-66-NH_2_

MOF	Initial rate (mM s^–1^)	Half-life (min)	TOF_all_ (s^–1^)	TOF_surface_ ^*a*^ (s^–1^)
UiO-66	0.010	35	0.0077	1.0
UiO-66-(OH)_2_	0.0063	60	0.0047	0.62
UiO-66-NO_2_	0.0070	45	0.0052	0.70
UiO-66-NH_2_	0.20	1	0.15	20

^*a*^400 nm particles of UiO-66 are synthesized. Due to the aperture size of UiO-66 and the relatively larger kinetic diameter of methyl-paraoxon, only the surface sites (*ca.* 0.75% of the catalyst loading) are catalytically active.

**Fig. 5:**
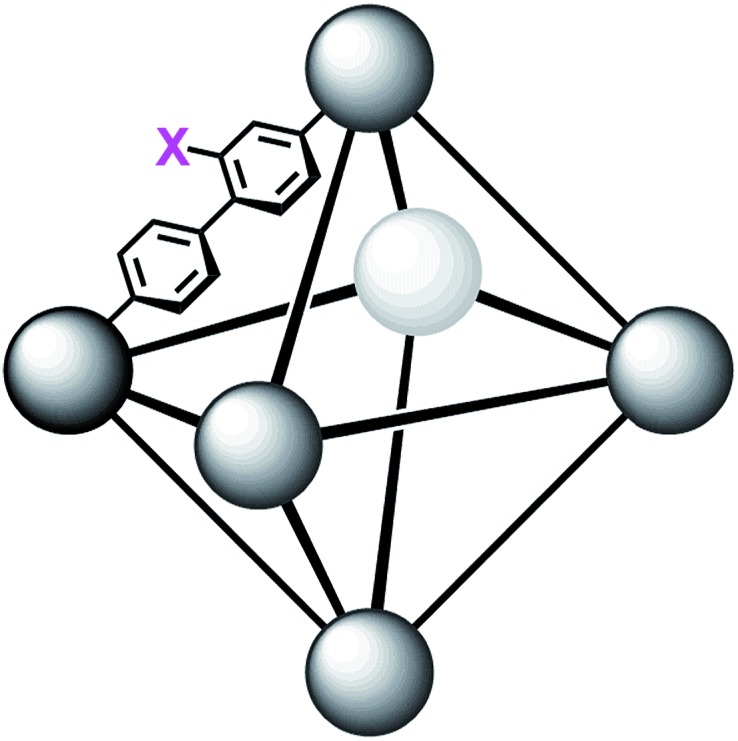
Connectivity of the octahedral pore in UiO-67 (right). X = H for UiO-67, X = NH_2_ for UiO-67-NH_2_, and X = N(CH_3_)_2_ for UiO-67-NMe_2_.

In order to further investigate the role of the amino moiety, we turned our attention to UiO-67 and its derivatives. In UiO-67 (Fig. 5), the functional groups point away from the node. Under these conditions, the amino moiety can only act as a Brønsted-base around the outer sphere of the active site.

**Fig. 6:**
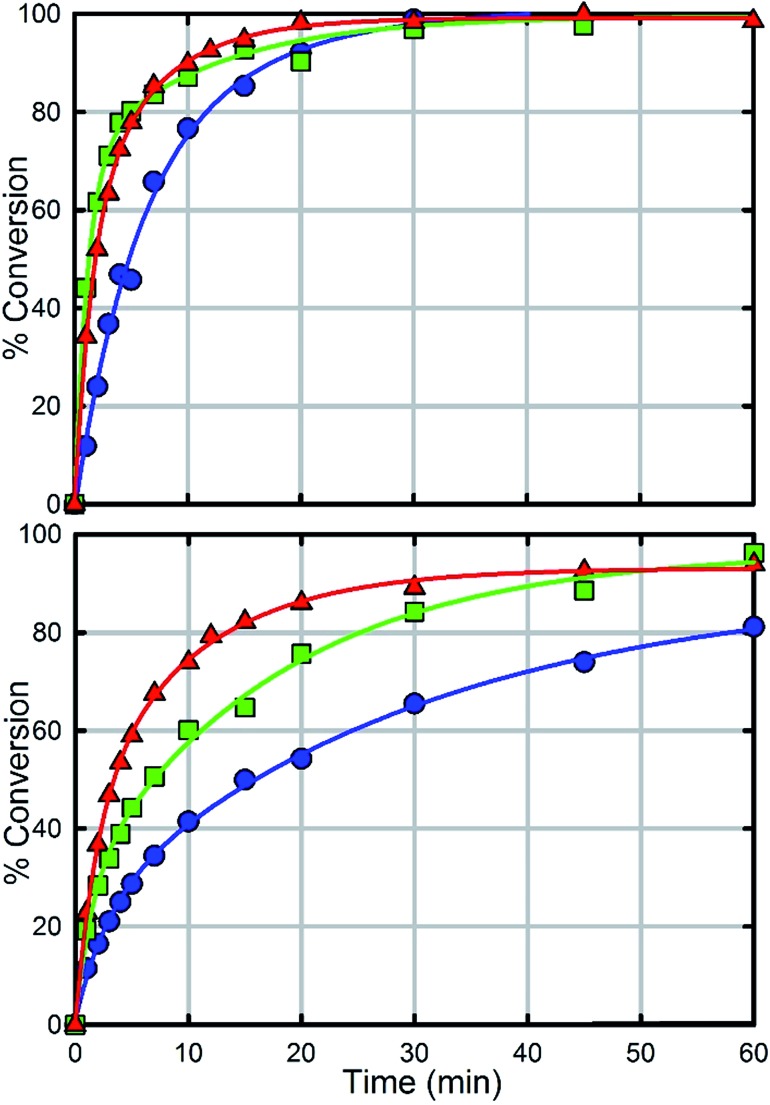
Hydrolysis rate of UiO-67 (blue), UiO-67-NH_2_ (red) and UiO-67-NMe_2_ (green) at full catalyst loading (1.5 μmole) with respect to UiO-66 (top) and at half catalyst loading (0.75 μmole) with respect to UiO-66 (bottom).

In comparison with UiO-66, UiO-67 (Fig. 6a) is a faster catalyst. We suspect that the longer distance between nodes prevents steric crowding around neighbouring nodes allowing for a more active material.^74^ Nevertheless, as with UiO-66, UiO-67-NH_2_ and UiO-67-NMe_2_ are faster than UiO-67 (Fig. 6, Table 2). Due to the fast hydrolysis of UiO-67, the rate enhancement (1.2 times) is not 20 times as observed between UiO-66 and UiO-66-NH_2_. Due to the fact that UiO-67-NH_2_ and UiO-67-NMe_2_ show nearly identical kinetic traces (Fig. 6), we suspect that a different part of the mechanism is now limiting. To illustrate this, the catalyst loading was decreased by a factor of two (Fig. 6b). The hydrolysis rate of UiO-67 decreased by a factor of 3.2 making UiO-67-NH_2_ 3.2 times faster than UiO-67. Furthermore, the hydrolysis rate of UiO-67-NMe_2_ decreased more than that of UiO-67-NH_2_ (Fig. 6b); these results support our hypothesis that UiO-67-NH_2_ and UiO-67-NMe_2_ are limited by another step in the catalytic cycle when compared to normal catalyst loadings.

**Table 2:** Initial rates and turn-over frequencies (TOFs) for UiO-67, UiO-67-NMe_2_, and UiO-67-NH_2_

MOF	Initial rate (mM s^–1^)	Half-life (min)	TOF_all_ (s^–1^)	TOF_surface_ ^*a*^ (s^–1^)
**Full catalyst loading**				
UiO-67	0.058	4.5	0.038	5.1
UiO-67-NMe_2_	0.078	2	0.052	7.0
UiO-67-NH_2_	0.067	2	0.044	6.0

**Half catalyst loading**				
UiO-67	0.018	15	0.024	3.2
UiO-67-NMe_2_	0.049	7	0.032	4.3
UiO-67-NH_2_	0.057	3.5	0.075	10

^*a*^400 nm particles of UiO-67 are synthesized; as with UiO-66, only the surface sites are expected to be catalytically active.

Thus, given the results above, we propose that the role of amino moiety in these derivatives is to act as a Brønsted-base whereby a proton is transferred to and from the base during the catalytic cycle. This is supported by the UiO-67-NMe_2_ hydrolysis which is slower than the UiO-67-NH_2_ hydrolysis likely due to the fact that the dimethylamino moiety is a stronger base and thus it is harder to deprotonate the moiety in order to regenerate the catalyst.

## Conclusions

The hydrolysis rate of six UiO-based MOFs was measured in order to probe the importance of various functionalities on the overall hydrolysis rate. We observed that only amino moieties were able to enhance the hydrolysis rate. A rate enhancement of up to 20 times was observed for UiO-66. Due to a careful comparison between UiO-66 and UiO-67 derivatives, we propose that the role of the amino moiety is to act like a Brønsted base, and subsequently a Brønsted acid during the catalytic cycle. With half-life of about 1 minute, UiO-66-NH_2_, is the fastest methyl-paraoxon hydrolysis MOF-based catalysts reported to date. With these results in hand, we are currently testing the generality of these design rules on other MOF topologies.
