# Supplementary material for: Exploiting parameter space in MOFs: a 20-fold enhancement of phosphate-ester hydrolysis with UiO-66-NH2
Source: Chem Sci. 2015 Feb 24;6(4):2286–91. doi: 10.1039/c4sc03613a (PMC5645779; doi:10.1039/c4sc03613a)
Supplement: Supplementary file 1 [file SC-006-C4SC03613A-s001.pdf]

Electronic Supplementary Material (ESI) for Chemical Science.
This journal is © The Royal Society of Chemistry 2015

[Supporting Information (SI) to accompany:]

Exploiting Parameter Space in MOFs: a 20-fold enhancement of phosphate-ester hydrolysis with UiO-66-NH_2

Michael J. Katz,[a] Su-Young Moon,[a] Joseph E. Mondloch,[a] M. Hassan Beyzavi,[a] Casey J. Stephenson,[a] Joseph T. Hupp,[a,b*] and Omar K. Farha.[a,c*]

[a]*Department of Chemistry and the International Institute for Nanotechnology, Northwestern University, 2145 Sheridan Road, Evanston, Illinois 60208−3113, United States*

[b]*Chemical Science and Engineering Division, Argonne National Laboratory, 9700 S. Cass Avenue, Argonne, Illinois 60439, United States.*

[c]*Department of Chemistry, Faculty of Science, King Abdulaziz University, Jeddah, Saudi Arabia*

** j-hupp@u.northwestern.edu; o-farha@northwestern.edu*

Table of Contents

Section S1: Powder x-ray diffractograms of UiO-66 and UiO-67 derivatives S2

Section S2: Nitrogen isotherms collected at 77 K for UiO-66 and UiO-67 derivatives S3

Section S3: Heterogeneity test S4

Section S1: Powder x-ray diffractograms of UiO-66 and UiO-67 derivatives

Powder x-ray diffractograms were collected on a Rigaku model ATXG diffractometer equipped with a Cu rotating anode x-ray source. This work made use of the J.B.Cohen X-Ray Diffraction Facility supported by the MRSEC program of the National Science Foundation (DMR-1121262) at the Materials Research Center of Northwestern University.

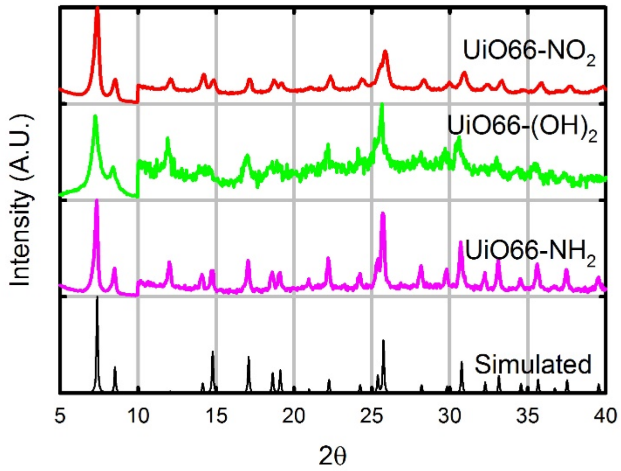

Figure S1: Powder x-ray diffractograms of UiO-66-NH_2, UiO-66-$(OH)_2$, and UiO-66-NO_2. The intensity from 10-40 ° is enhanced 10x for clarity.

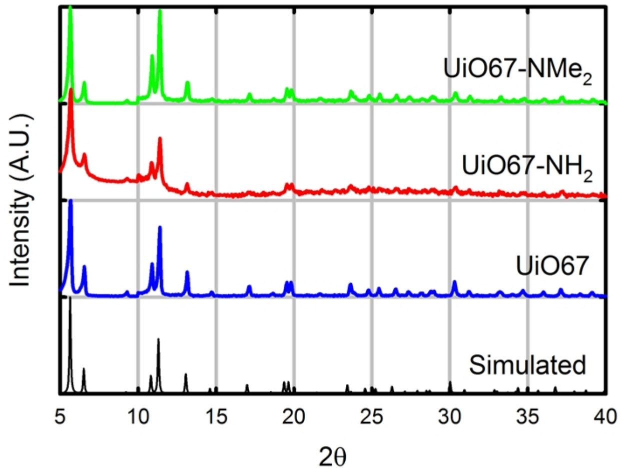

Figure S2: Powder x-ray diffractograms of UiO-67, UiO-67-NH_2, and UiO-66-NMe_2. The intensity from 10-40 ° is enhanced 10x for clarity.

Section S3: Nitrogen isotherms collected at 77 K for UiO-66 and UiO-67 derivatives

N_2 adsorption and desorption isotherm measurements were performed on a Micromeritics Tristar II 3020 (Micromeritics, Norcross, GA) at 77K. Before each isotherm, samples were activated either via supercritical CO_2 drying (UiO-67 and derivatives) or by heating for 3 hours (UiO-66 and derivatives) under high vacuum on a Smart-Vac Prep at 150 °C (Micromeritics, Norcross, GA). Between 30 and 100 mg of material was used for each measurement. Data was analyzed using the ASAP 2020 software (Micromeritics, Norcross, GA). All gases used were Ultra High Purity Grade 5 as obtained from Airgas Specialty Gases (Chicago, IL).

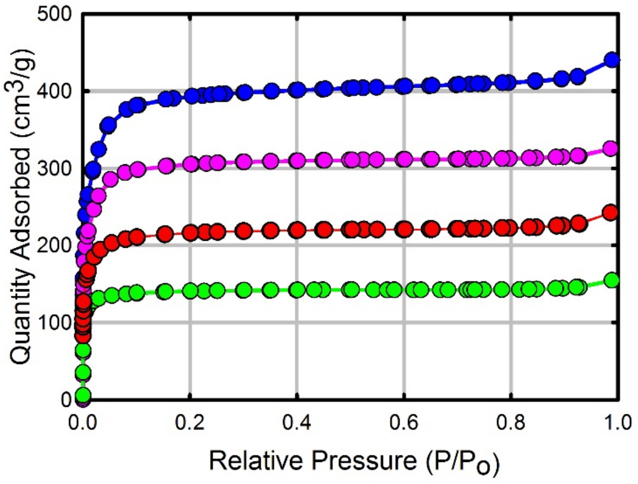

Figure S3: N_2 isotherms collected at 77 K for UiO-66 (blue), UiO-66-NH_2 (pink), UiO-66-NO_2 (red), and UiO-66-$(OH)_2$ (green).

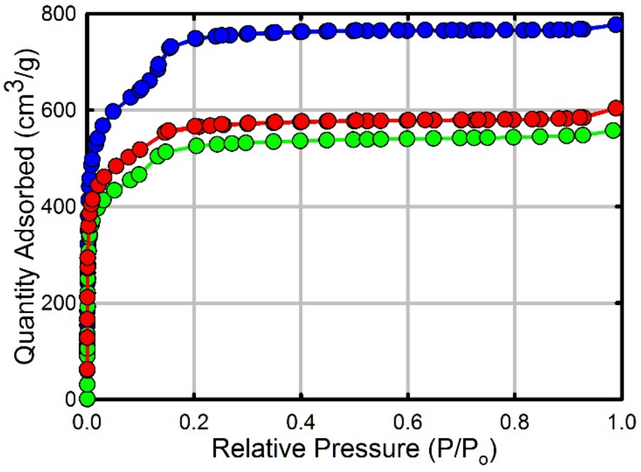

Figure S4: N_2 isotherms collected at 77 K for UiO-67 (blue), UiO-67-NH_2 (red), and UiO-67-NMe_2 (green).

Section S3: Heterogeneity test

In order to ensure that the fast hydrolysis rate observed for UiO-66-NH_2 is due to the MOF and not leaching of zirconium into solution, the hydrolysis rate was monitored until 1.8 min after which the reaction mixture was filtered through a 0.2 μm filter; no further catalysis was observed up to 60 min as shown in Figure S5. Additionally, ICP-AES of the filtered solution showed no zirconium present in solution.

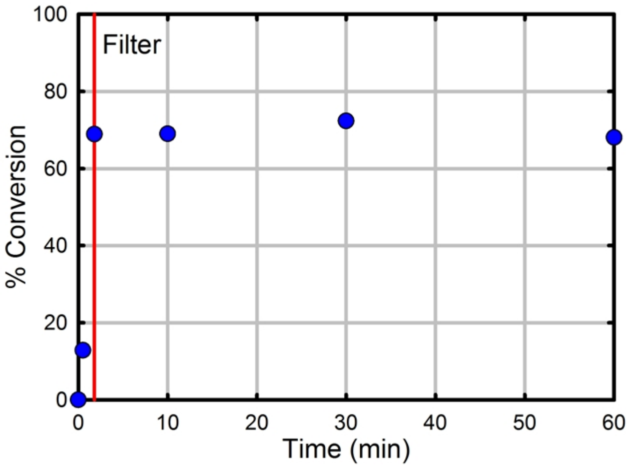

Figure S5: Heterogeneity test of UiO-66-NH_2. A catalyst sample was filtered at 1.8 min (red line). Note, no additional catalysis is observed after filtration.
